# Corrigendum to ‘Health impacts of daily weather fluctuations: Empirical evidence from COVID-19 in U.S. counties’ [J. Environ. Manag. (2021) 112662]

**DOI:** 10.1016/j.jenvman.2024.123687

**Published:** 2025-01

**Authors:** Lotanna Emediegwu

**Affiliations:** Manchester Metropolitan University, UK

The authors regret < that an error was identified in the first sentence of the Introduction. Specifically, COVID-19 was discovered in December 2019 and not 2020, as mistakenly reported.>.

The authors would like to apologise for any inconvenience caused.

